# Vitamin D status affects the relationship between lipid profile and high-sensitivity C-reactive protein

**DOI:** 10.1186/s12986-020-00455-x

**Published:** 2020-07-14

**Authors:** Dan Jin, Dao-Min Zhu, Hong-Lin Hu, Meng-Nan Yao, Wan-Jun Yin, Rui-Xue Tao, Peng Zhu

**Affiliations:** 1 Department of Maternal, Child and Adolescent Health, School of Public Health, Anhui Medical University, Hefei, China; 2 MOE Key Laboratory of Population Health Across Life Cycle, Hefei, China; 3 NHC Key Laboratory of Study on Abnormal Gametes and Reproductive Tract, Anhui Medical University, Hefei, China; 4 Anhui Provincial Key Laboratory of Population Health and Aristogenics, Anhui Medical University, Hefei, China; 5 Department of Sleep Disorders, Hefei Fourth People’s Hospital, Hefei, China; 6 Department of Endocrinology, the First Affiliated Hospital of Anhui Medical University, Hefei, China; 7 Department of Gynecology and Obstetrics, Hefei First People’s Hospital, Hefei, China

**Keywords:** 25(OH)D, Hs-CRP, Lipid profile, Pregnancy

## Abstract

**Background:**

The biological pathways through which vitamin D is involved in the regulation of systemic inflammation remain largely unknown.

**Objective:**

The objective of this study was to evaluate the role of vitamin D status on the relationship between lipid profile and high-sensitivity C-reactive protein (hs-CRP) in pregnant women.

**Design:**

Serum 25-hydroxyvitamin D (25(OH)D), hs-CRP, and indicators of lipid profiles (total cholesterol, TC; triglyceride, TG; high-density lipoprotein cholesterol, HDL-C; low-density lipoprotein cholesterol, LDL-C), were measured in 2479 pregnant women during the second trimester. Potential confounding including maternal sociodemographic characteristics, perinatal health status, diet, and lifestyle was prospectively collected. Multiple regression models and cubic models were used to evaluate the associations.

**Results:**

There was a significant non-linear relationship between lipid profile (TC, TG, HDL-C, LDL-C) and hs-CRP (*P* < 0.05). Increased serum 25(OH)D was significantly associated with decreasing TC, TG, HDL-C, LDL-C, and hs-CRP levels. Compared with medium levels of lipids group, pregnant women with higher levels of TC or TG have higher levels of hs-CRP, and pregnant women with lower levels of TC, HDL-C or LDL-C also have higher levels of hs-CRP in the vitamin D deficient group, and there was a significant correlation between low levels of TG and decreased hs-CRP (adjusted *β* for TG: -0.063, 95%*CI*: − 0.120,-0.007) in the non-vitamin D deficient group. Mediators that had appreciable shares of the associations between 25(OH)D and hs-CRP was TG (10.2% of the association; *β* = − 0.011; total indirect effect: 95% *CI*: − 0.019, − 0.002). The cubic model suggested that a steep increase in the adjusted regression coefficient of lipid with hs-CRP up to 50 nmol/L of 25(OH)D, and the highest adjusted regression coefficients were observed in pregnant women with 25(OH)D above 50 nmol/L.

**Conclusion:**

Our findings suggest that high levels of vitamin D during pregnancy may improve lipid profile levels and inhibit elevated hs-CRP induced by high lipid metabolism.

## Introduction

Increasing evidence suggests that dyslipidemia is often associated with elevated levels of inflammatory markers (e.g., tumor necrosis factor-β, interleukin 6 and hs-CRP) and is closely relationship with metabolic disease including obesity, type 2 diabetes, and cardiovascular disease [1–3], this metabolically triggered inflammation is called metabolic inflammation [4]. Although physiological changes of lipids during pregnancy may have specific causes for fetus development [5], studies revealed that some factors such as obesity [6], physical activity [7], diet and lifestyle [8] negatively influence lipid profile increase, and it is possible to further enhance the systemic inflammatory response, causing the occurrence of metabolic inflammation. Metabolic inflammation may play an important role in the mechanism of gestational diabetes [9].

Vitamin D may have immunomodulatory effects, serum 25(OH)D concentrations were inversely related to systemic inflammatory markers such as hs-CRP [10, 11]. However, biological pathways through which vitamin D participates in the regulation of systemic inflammation remain largely unknown. Previous studies have shown that vitamin D deficiency exacerbates dyslipidemia. Therefore, vitamin D deficiency may further enhance the systemic inflammatory response through aggravating dyslipidemia. To date, some studies on the associations among vitamin D, lipid profile and systemic inflammatory response are mostly from the general population. It is not clear whether the conclusion of the general population was applicable to pregnant women. In addition, the role of pregnancy-related vitamin D on lipid profile and systemic inflammatory markers remains inconsistent during pregnancy from several small sample studies [12–14]. Therefore, more high-quality research is needed to clarify.

In this study, based on a larger sample, we aimed to assess the role of vitamin D during pregnancy on the relationship between lipid profile and hs-CRP, stratifications by vitamin D status was performed to further examine the associations.

## Methods

### Study design and participants

This study was conducted based on a prospective birth cohort. A total of 6712 pregnant women aged 18–45, with gestational ages from 14 to 27 weeks, were recruited in Hefei (latitude 32), China, between March 2015 and September 2018. At the time of enrollment, participants completed a self-designed structured questionnaire including sociodemographic characteristics, perinatal health status, diet, and lifestyle. Midwives or study nurses collected the pregnant anthropometric details and blood samples. The exclusion criteria included: multiple pregnancies, severe pregnancy complications (e.g., heart failure, severe anemia, gestational hypertension, etc.), abnormal function in liver, kidney or thyroid, artificially assisted reproduction, incomplete follow-up data during pregnancy, a toll of 5456 effective research subjects was obtained. We randomly selected half of the samples (2728 samples) were assayed for serum 25(OH)D, lipid profile and hs-CRP, and 249 samples in which there at least one index lower than the lower limit of detection was excluded. Finally, there were 2479 pregnant women in our study (Supplemental Figure 1). Written informed consent was obtained from all pregnant women, and the research protocol was approved by the Ethics Committee of Anhui Medical University (batch number: 2015002).

### Blood samples

Blood samples collection (5 mL) was performed between 7:00 am and 9:00 am by a trained technician (nurse) after 8–12 h of overnight fasting. Serum samples were immediately centrifuged (3000 rpm for 5 min) and stored at − 80 °C until analysis.

Serum 25(OH)D and hs-CRP were measured by using the Electrochemical Luminescence Detection Kit for Roche E601 (Sandhofer, Mannheim, Germany) and the enzyme-linked immunosorbent assay (ELISA) kits (Cusabio Biotech, Wuhan, China) according to manufacturer’s instructions. Interclass and intraclass coefficients of variation were less than 10%.

The concentrations of serum TC, TG, HDL-C, and LDL-C were evaluated using an automatic analyzer (Beckman Coulter, Brea, CA, USA) and a commercial kit (Leadman, Beijing, China). TG level was tested by the glycerol-3-phosphate oxidase peroxidase-anti-peroxidase (PAP) method. TC was measured using the cholesterol oxidase PAP method. HDL-C and LDL-C were measured by direct measurement. The coefficient of variation (CV) for inter- and intra-assay was 4 and 3% for TC, 6 and 5% for TG, 6 and 4% for HDL-C, 4 and 3% for LDL-C.

Serum 25(OH)D concentrations were classified as vitamin D deficiency (< 50 nmol/L) and non-vitamin D deficiency (≥50 nmol/L) by the Institute of Medicine (IOM) [15]. In the absence of pregnancy-specific reference ranges, in this study, the lipid profile (TC, TG, HDL-C, and LDL-C) grouped by the equal quartile method (P25, P50, P75) were combined into the following three groups from the perspective of exploration: low level (<P25), medium-level (P25-P75) and high level (≥P75), the specific number of participants in each category was shown in Table 2.

### Potential confounders

The following potential confounding was obtained from interviews or medical records. Maternal sociodemographic characteristics included age(years), an education level (≥9 and < 9 years of completed schooling), income (less than 4000 and greater than 4000 RMB Yuan/month). Perinatal health status included pre-pregnancy body mass index (Pre-pregnancy BMI, based on self-reported weight divided by the measured squared height at prenatal visit), systolic and diastolic blood pressure (mmHg), parity (nulliparous or multiparous). Diet and lifestyle included the frequency of physical exercise for no less than 10 min per week (< 3 and ≥ 3 days/week), the time to sedentary time every day (< 4 and ≥ 4 h/day), paternal alcohol (yes or no ) and smoking (less or more than 6 cigarettes/day) in the last 3 months. Frequency of food intake (e.g., mike, soy product, and dessert intake), fish oil, and multivitamin supplement in the past week. The season was designated as summer (June, July, August), autumn (September, October, November), winter (December, January, February) or spring (March, April, May).

### Statistical analysis

The characteristics and laboratory statistics of 2479 pregnant women were described as the percentage or means (standard deviations, SD). In this study, non-normally distributed variables of hs-CRP were logarithmically transformed for further regression analysis.

Non-linear relations of lipid profile and hs-CRP have fitted the data in non-linear regression models using a cubic polynomial fitting. We divided the lipid metabolism index into three groups (low level: <P25; medium-level: P25-P75; high level:≥P75) based on the observed non-linear relationship. Multiple linear regression models were used to estimate associations between lipid profile status and hs-CRP with adjusting for potential confounding factors.

Crude and adjusted regression coefficients were generated for the associations of 25(OH)D with lipid profile and hs-CRP by using multiple linear regression analyses. Stratifying by vitamin D status (25(OH)D < 50 nmol/L and 25(OH)D ≥ 50 nmol/L), we further assessed the associations between lipid profile and hs-CRP by using multiple linear models. Using cubic fitting to test the association of serum 25(OH)D levels and adjusted regression coefficients for lipid profile and inflammatory marker hs-CRP.

Supplemental Figure 2 shows the direct and indirect effects from 25(OH)D to hs-CRP through intervening variables (TC, TG, HDL-C, and LDL-C) by using a path analysis model (Analysis of Moment Structures27). The bootstrap method (sample = 5000) was used to estimate the mediating effect, and the indirect effect was obtained by bias-corrected CI estimation. Statistical analyses were performed using SPSS 21.0 software (Statistical Package for the Social Sciences version 21.0, IBM Corp: Armonk, NY, USA).

## Results

### Sociodemographic and laboratory characteristics of the participants

The study included 2479 pregnant women, the mean age of the study population was 29.3 ± 4.2 years, the percentage of more frequency of maternal education (≥9 years) was 60.7% (*n* = 1505). The mean pre-pregnancy BMI was 21.2 ± 2.8 kg/m^2^, and 42.3% (*n* = 1049) of participants were multipara. The mean of serum 25(OH)D and hs-CRP concentrations were 40.08 nmol/L (SD:16.69, range:10.00–114.00) and 3.03 mg/L (SD:2.07, range:0.20–10.00), the percentage of the participants who had vitamin D deficiency was 75.6% (*n* = 1875). The mean of serum TC, TG, HDL-C, and LDL-C concentrations were 5.99 mmol/L (SD:1.01, range:2.90–9.71), 2.61 mmol/L (SD:1.02, range:0.80–9.87), 2.02 mmol/L (SD:0.35, range:0.76–3.67), and 2.85 mmol/L (SD:0.70, range:0.70–7.42), respectively (Table 1).

**Table 1:** Characteristics of the study population in the second trimester^a^

Characteristics	All(***n*** = 2479)	25(OH)D ≥ 50 nmol/L(***n*** = 604)	25(OH)D<50 nmol/L(***n*** = 1875)
	Mean ± SD or n(%)	Mean ± SD or n(%)	Mean ± SD or n(%)
25(OH)D status	40.1 ± 16.7	**63.2** ± 13.0	**32.6** ± 9.1
**Sociodemographic characteristics**			
Maternal age(years)	29.3 ± 4.2	29.2 ± 4.1	29.3 ± 4.2
Maternal education(< 9 years)	974(39.3)	212(35.1)	675(36.0)
Maternal income(< 4000 RMB yuan/month)	1657(66.8)	403(66.7)	1254(66.9)
Entered in summer or autumn	1280(51.6)	257(42.5)	942(50.2)*
**Perinatal health status**			
Pre-pregnancy BMI(kg/m^2^)	21.2 ± 2.8	21.0 ± 2.7	21.3 ± 2.8*
BMI(kg/m^2^)	24.2 ± 2.9	23.8 ± 2.7	24.3 ± 2.9*
Systolic blood pressure(mmHg)	109.8 ± 9.5	109.5 ± 9.9	109.9 ± 9.4
Diastolic blood pressure(mmHg)	68.7 ± 7.6	68.6 ± 8.8	68.7 ± 7.3
Blood glucose	4.6 ± 0.4	4.6 ± 0.4	4.6 ± 0.4
Multipara	1049(42.3)	264(43.7)	785(41.9)
**Diet and Lifestyle**			
Sedentary time(≥4 h/day)	1773(71.5)	409(67.7)	511(27.3)*
Physical activities(<3 days/week)	1453(58.6)	351(58.1)	1102(58.8)
Paternal alcohol consumption(no times/week)	1647(66.4)	409(67.7)	1238(66.0)
Paternal smoking(≤6 cigarettes/day)	1980(79.9)	481(79.6)	1499(79.9)
fish oil supplement(≤3 times/week)	2420(97.6)	573(94.9)	1874(98.5)*
multivitamin supplement(≤3 times/week)	2264(91.3)	495(82.0)	1769(94.3)*
Milk intake(≥3 times/week)	1417(57.2)	375(62.1)	1042(55.6)*
Soy product intake(≥3 times/week)	1146(46.2)	293(48.5)	853(45.5)
Dessert intake(≥3 times/week)	442(17.8)	88(14.6)	354(18.9)*
**Laboratory testing indicators**			
Hs-CRP(mg/L)	3.0 ± 2.1	2.7 ± 1.8	3.1 ± 2.1
TC(mmol/L)	6.0 ± 1.0	5.8 ± 1.0	6.0 ± 1.0
TG(mmol/L)	2.6 ± 1.0	2.5 ± 1.0	2.6 ± 1.0
HDL-C(mmol/L)	2.0 ± 0.4	2.0 ± 0.3	2.0 ± 0.4
LDL-C(mmol/L)	2.8 ± 0.7	2.8 ± 0.7	2.9 ± 0.7

a.*25(OH)D* 25-Hydroxyvitamin D; *hs-CRP* High-sensitivity C-reactive protein; *TC* Total cholesterol; *TG* triglyceride; *HDL-C* High-density lipoprotein-cholesterol; *LDL-C* Low-density lipoprotein-cholesterol; * Indicates P < 0.05

### Association of lipid profile and hs-CRP levels in the second trimester

**Fig. 1:**
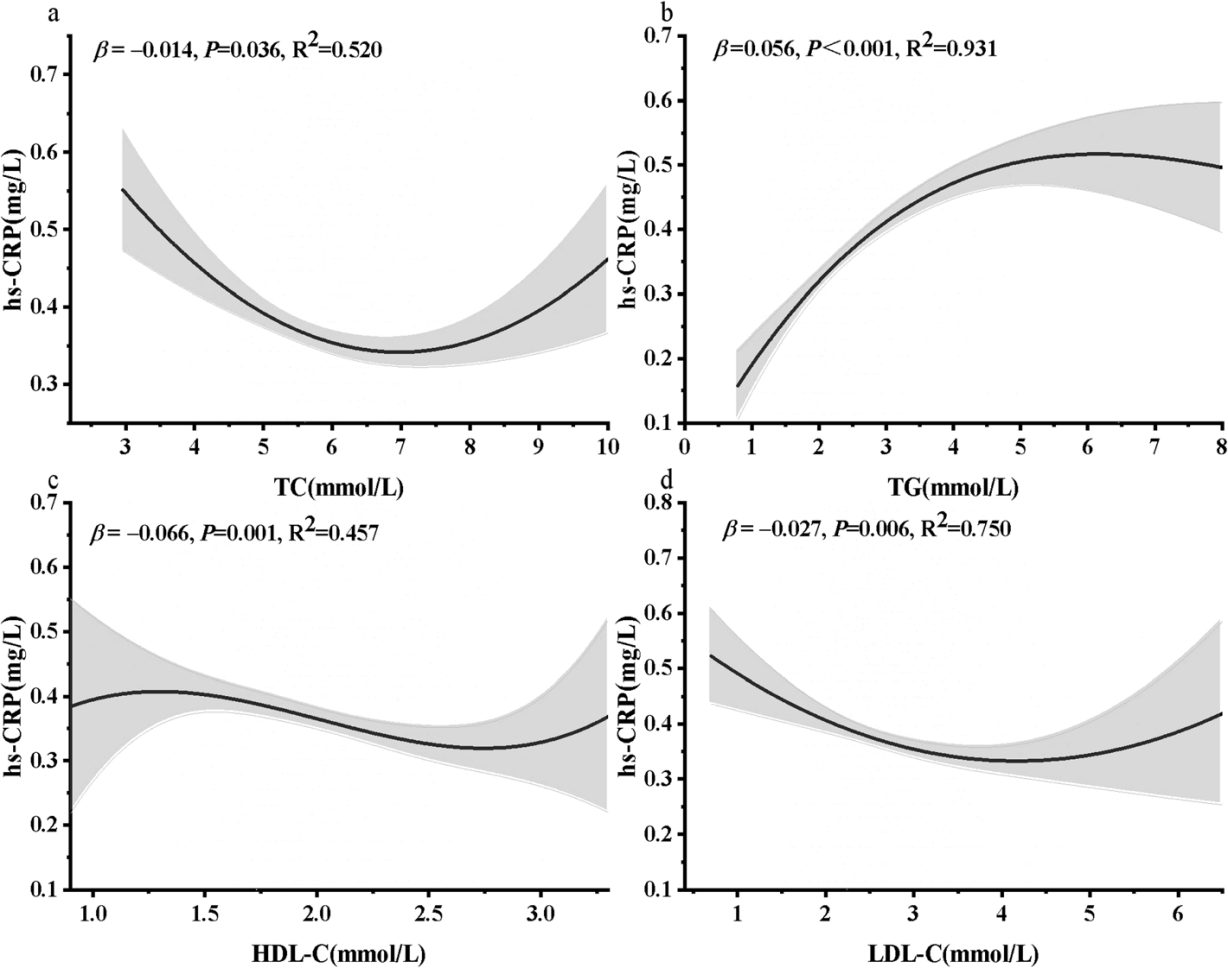
Non-linear relationship between lipid profile and hs-CRP in the second trimester

Polynomial fitting revealed a significant non-linear correlation between lipids level (TC: *R*^*2*^ = 0.520, *P* = 0.036; TG: *R*^*2*^ = 0.931, *P* <0.001; HDL-C: *R*^*2*^ = 0.457, *P* = 0.001; LDL-C: *R*^*2*^ = 0.750, *P* = 0.006) and hs-CRP, respectively (Fig. 1). The U-shaped nature of the association of TC and LDL-C with hs-CRP was confirmed with cubic-fitting model. After adjustment for confounding, compared with the medium level of lipid profile group, the hs-CRP levels in the low (adjusted *β*: 0.040, 95%CI: 0.009,0.071) or high (adjusted *β*: 0.043, 95%CI: 0.012,0.074) level group of TC, high leaves of TG (adjusted *β*: 0.040, 95%CI: 0.010,0.070), or low LDL-C (adjusted*β*: 0.050, 95%CI: 0.020,0.080) levels were significantly elevated, while hs-CRP was significantly reduced only at low TG (adjusted*β*: -0.077, 95%CI:-0.108,-0.047) levels. However, there was no significant association between HDL-C and hs- CRP (Table 2).

**Table 2:** Association of lipid profile and hs-CRP levels in the second trimester^a^

Groups	n	Hs-CRP (mg/L) level		
		Mean ± SD	***Adjustedβ*** (95%***CI***)^**b**^	***P***
**TC status**				
<P25	615	3.26 ± 2.18	0.040(0.009,0.071)	0.011
P25 ~ P75	1238	2.89 ± 1.98	**Ref**^**c1**^	
≥ P75	626	3.13 ± 2.12	0.043(0.012,0.074)	0.006
**TG status**				
<P25	633	2.49 ± 1.88	-0.077(−0.108,-0.047)	<0.001
P25 ~ P75	1230	3.05 ± 2.01	**Ref**^**c2**^	
≥ P75	616	3.59 ± 2.23	0.040(0.010,0.070)	0.010
**HDL-C status**				
<P25	599	3.27 ± 2.13	0.018(−0.013,0.049)	0.256
P25 ~ P75	1271	3.02 ± 2.07	**Ref**^**c3**^	
≥ P75	609	2.86 ± 2.00	−0.014(− 0.044,0.017)	0.388
**LDL-C status**				
<P25	621	3.37 ± 2.28	0.050(0.020,0.080)	0.001
P25 ~ P75	1244	2.86 ± 1.88	**Ref**^**c4**^	
≥ P75	614	3.08 ± 2.18	0.019(−0.012,0.049)	0.235

a.*25(OH)D* 25-Hydroxyvitamin D; *hs-CRP* High-sensitivity C-reactive protein; *TC* Total cholesterol; *TG* Triglyceride; *HDL-C* High-density lipoprotein-cholesterol; *LDL-C* Low-density lipoprotein-cholesterol;

b. Sociodemographic characteristics (maternal age, education, income, season), perinatal health status(pre-pregnancy BMI, systolic blood pressure and diastolic blood pressure), lifestyle(sedentary time, physical activities, paternal alcohol and smoking consumption, fish oil supplement, multivitamin supplement, milk intake, soy product intake and dessert intake), and 25(OH)D

c. “Ref^c1^” indicates P25 ≤ TC<P75 as a reference group; “Ref^c2^” indicates P25 ≤ TG<P75 as a reference group; “Ref^c3^” indicates P25 ≤ HDL-C<P75 as a reference group; “Ref^c4^” indicates P25 ≤ LDL-C<P75 as a reference group

### Association of 25(OH)D status with lipid profile and hs-CRP in the second trimester

Table 3 shows the relationship between 25(OH)D status with lipid profile and hs-CRP in the second trimester of pregnancy. Adjusted multiple linear regression analyses revealed a significant negative correlation between serum 25(OH)D with TC, TG, HDL-C, LDL-C, and hs-CRP. Compared with vitamin D deficiency group, the levels of TC, TG, HDL-C, LDL-C and hs-CRP in the non-vitamin D deficiency group significant decrease by 8.690 mg/dL (95%CI: 5.046,12.333), 13.254 mg/dL (95%CI: 4.963,21.544), 1.910 mg/dL (95%CI: 0.644,3.176), 4.933 mg/dL (95%CI: 2.409,7.458), and 0.034 mg/L (95%CI: 0.004,0.064), respectively.

**Table 3:** Association of vitamin D status with lipid profile and hs-CRP in the second trimester^a^

Variables	***β*** (95%***CI***)	Vitamin D Status	
		25(OH)D ≥ 50 nmol/L	25(OH)D<50 nmol/L
**TC(mg/dL)**			
Model 1^b^	−0.212(− 0.304,-0.120)*	−8.384(−11.965,-4.803)*	**Ref**^**d**^
Model 2^c^	− 0.248(− 0.344,-0.152)*	−8.690(− 12.333,-5.046)*	**Ref**
**TG(mg/dL)**			
Model 1^b^	−0.477(− 0.689,-0.265)*	−13.630(−21.916,-5.344)*	**Ref**
Model 2^c^	−0.468(− 0.687,-0.250)*	−13.254(− 21.544,-4.963)*	**Ref**
**HDL-C(mg/dL)**			
Model 1^b^	−0.018(− 0.050,0.014)	−1.307(−2.554,-0.060)*	**Ref**
Model 2^c^	− 0.045(− 0..078,-0.011)*	−1.910(−3.176,-0.644)*	**Ref**
**LDL-C(mg/dL)**			
Model 1^b^	−0.171(− 0.234,-0.107)*	−5.360(−7.831,-2.888)*	**Ref**
Model 2^c^	− 0.176(− 0.242,-0.109)*	−4.933(− 7.458,-2.409)*	**Ref**
**Hs-CRP(mg/L)**			
Model 1^b^	− 0.002(− 0.003,-0.001)*	− 0.050(− 0.080,-0.019)*	**Ref**
Model 2^c^	−0.002(− 0.002,-0.001)*	−0.034(− 0.064,-0.004)*	**Ref**

a.*25(OH)D* 25-Hydroxyvitamin D; *hs-CRP* High-sensitivity C-reactive protein; *TC* Total cholesterol; *TG* Triglyceride; *HDL-C* High density lipoprotein-cholesterol; *LDL-C* Low density lipoprotein-cholesterol; Unit conversion factors: TC, HDL-C, LDL-C((mg/dL) × 0.0259 = mmol/L); TG((mg/dL) × 0.0113 = mmol/L);

b.Model 1 unadjusted confounding factor;

c.Model 2 controls confounding factors based on Model 1, including adjusted for sociodemographic characteristics (maternal age, education, income, season), perinatal health status(pre-pregnancy BMI, systolic blood pressure and diastolic blood pressure), lifestyle(sedentary time, physical activities, paternal alcohol and smoking consumption, fish oil supplement, multivitamin supplement, milk intake, soy product intake and dessert intake), hs-CRP or lipid profile

d. “Ref^d^” indicates 25(OH)D<50 nmol/L as a reference group

* Indicates *P* < 0.05

### Relationship between lipid profile and hs-CRP in different levels of vitamin D

Stratification by 25(OH)D level was performed to further explore the association of lipid profile and hs-CRP (Table 4). Compared with medium levels of lipids group, hs-CRP levels are higher in pregnant women with high TC or TG levels, and pregnant women with lower levels of TC, HDL-C or LDL-C also have higher hs-CRP levels in the vitamin D deficient group. However, in the non-vitamin D deficient group, a higher lipid profile was not associated with increased hs-CRP levels. Additionally, there was a significant association of lower levels of TG with decreased hs-CRP.

**Table 4:** Relationship between lipid profile and hs-CRP in different levels of vitamin D^a^

Groups	n(%)	Hs-CRP (mg/L) levels		
		Mean ± SD	Adjusted***β***(95%***CI***)^**b**^	***P***
**25(OH)D < 50 nmol/L**				
TC<P25	435(23.2)	3.34 ± 2.27	0.038(0.001,0.074)	0.042
P25 ≤ TC < P75	944(50.3)	2.99 ± 2.06	**Ref**^**c1**^	
TC ≥ P75	496(26.5)	3.18 ± 2.19	0.041(0.005,0.076)	0.024
TG<P25	440(23.5)	2.54 ± 1.95	−0.085(− 0.121,-0.048)	< 0.001
P25 ≤ TG < P75	940(50.1)	3.12 ± 2.10	**Ref**^**c2**^	
TG ≥ P75	495(26.4)	3.65 ± 2.26	0.048(0.014,0.083)	0.006
HDL-C<P25	443(23.6)	3.42 ± 2.25	0.038(0.002,0.074)	0.040
P25 ≤ HDL-C < P75	964(51.4)	3.06 ± 2.13	**Ref**^**c3**^	
HDL-C ≥ P75	468(25.0)	2.97 ± 2.06	−0.002(−0.033,0.037)	0.909
LDL-C<P25	432(23.0)	3.44 ± 2.40	0.050(0.014,0.085)	0.006
P25 ≤ LDL-C < P75	955(50.9)	2.94 ± 1.93	**Ref**^**c4**^	
LDL-C ≥ P75	488(26.1)	3.20 ± 2.28	0.025 (−0.010,0.059)	0.166
**25(OH)D ≥ 50 nmol/L**				
TC<P25	180(29.8)	2.96 ± 1.89	0.016(−0.042,0.074)	0.582
P25 ≤ TC < P75	294(48.7)	2.54 ± 1.66	**Ref**^**c1**^	
TC ≥ P75	130(21.5)	2.86 ± 1.81	0.058(−0.010,0.127)	0.096
TG<P25	193(32.0)	2.38 ± 1.72	−0.063(,-0.120,-0.007)	0.029
P25 ≤ TG < P75	290(48.0)	2.83 ± 1.71	**Ref**^**c2**^	
TG ≥ P75	121(20.0)	3.07 ± 1.92	0.018 (−0.045,0.082)	0.573
HDL-C<P25	156(25.8)	2.74 ± 1.63	−0.046(− 0.106,0.014)	0.113
P25 ≤ HDL-C < P75	307(50.8)	2.86 ± 1.87	**Ref**^**c3**^	
HDL-C ≥ P75	141(23.4)	2.46 ± 1.69	−0.058(−0.121,0.005)	0.070
LDL-C<P25	189(31.3)	3.06 ± 1.94	0.034(−0.023,0.090)	0.240
P25 ≤ LDL-C < P75	289(47.8)	2.57 ± 1.65	**Ref**^**c4**^	
LDL-C ≥ P75	126(20.9)	2.63 ± 1.72	0.006(−0.060,0.063)	0.869

a.*25(OH)D* 25-Hydroxyvitamin D; *hs-CRP* High-sensitivity C-reactive protein; *TC* Total cholesterol; *TG* Triglyceride; *HDL-C* High-density lipoprotein-cholesterol; *LDL-C* Low-density lipoprotein-cholesterol;

b.Adjusted for sociodemographic characteristics (maternal age, education, income, season), perinatal health status(pre-pregnancy BMI, systolic blood pressure and diastolic blood pressure), lifestyle(sedentary time, physical activities, paternal alcohol and smoking consumption, fish oil supplement, multivitamin supplement, milk intake, soy product intake and dessert intake)

c. “Ref^c1^” indicates P25 ≤ TC<P75 as a reference group; “Ref^c2^” indicates P25 ≤ TG<P75 as a reference group; “Ref^c3^” indicates P25 ≤ HDL-C<P75 as a reference group; “Ref^c4^” indicates P25 ≤ LDL-C<P75 as a reference group

HDL-C was not included in path analysis because no significant association between 25(OH)D and HDL-C was found. For the comparative fit index, normal fit index, and the root mean square error of approximation of our path model, values were 0.781, 0.870, 0.001, respectively, reflecting an acceptable fit. Supplemental Figure 2 shows that a 1 nmol/L increase in 25(OH)D was associated with a 20%, a 10%, and a 23% decrease in TC, TG and LDL-C concentrations (*P* < 0.05). If the TG concentration increased by 1%, we would expect hs-CRP to increase by 0.02 mg/L (*P* < 0.05). Mediators that had appreciable shares of the associations between 25(OH)D and hs-CRP was TG (10.2% of the association; β = − 0.011; total indirect effect: 95% CI: − 0.019, − 0.002).

### The role of 25(OH)D in the relationship between lipid profile and hs-CRP in the second trimester

**Fig. 2:**
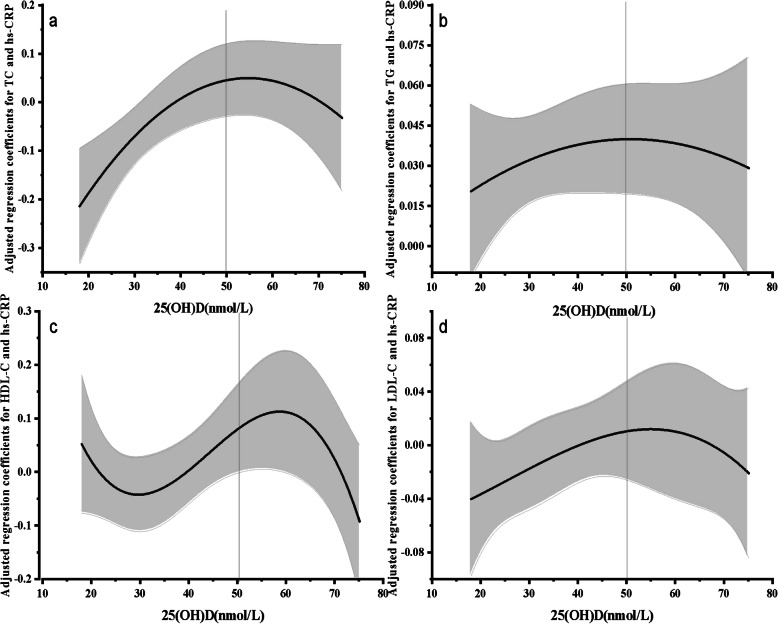
The role of vitamin D in the relationship between lipid profile and inflammation in the second trimester. a.25(OH)D, 25-hydroxyvitamin D; hs-CRP, high-sensitivity C-reactive protein; TC, total cholesterol; TG, triglyceride; HDL-C, high density lipoprotein-cholesterol; LDL-C, low density lipoprotein-cholesterol; Note: The ordinates denote the adjusted regression coefficient of the multiple linear regression of lipid profile and hs-CRP, and the abscissa denote the 25(OH)D level in the second trimester; the solid black line denote the correlation between vitamin D levels with lipid profile and hs-CRP in the second trimester, and the shaded area indicates a 95% confidence interval. The closer the distance of the solid line from the X axis, the stronger the correlation strength between the two. Adjusted for sociodemographic characteristics (maternal age, education, income, season, parental diabetes and parental rheumatism), perinatal health status(pre-pregnancy BMI, systolic blood pressure and diastolic blood pressure), lifestyle(sitting or lying time, physical activities, paternal alcohol and smoking consumption, fish oil supplement, multivitamin supplement, milk intake, soy product intake and dessert intake)

We further assessed the role of 25(OH)D status in the relationship between lipid profile and hs-CRP in the second trimester by cubic model, the solid black line shows the best fit analysis (TC: *R*^*2*^ = 0.68, *P* = 0.03; TG: *R*^2^ = 0.14, *P* = 0.81; HDL- C: *R*^2^ = 0.63, *P* = 0.03; LDL-C: *R*^2^ = 0.41, *P* = 0.21) (Fig. 2). After adjusting for confounds, this model suggested a steep increase in the adjusted regression coefficient of lipid with hs-CRP up to 50 nmol/L of 25(OH)D, and the highest adjusted regression coefficients were observed in pregnant women with 25(OH)D above 50 nmol/L.

## Discussion

In the present study, we first evaluated the relationship between lipid profile and hs-CRP in a large sample. Results showed that lipid metabolic indicators were significantly associated with elevated inflammation during pregnancy, and pregnant women with high levels of TC and TG had higher hs-CRP. Notably, there was a significant non-linear relationship between lipid profile (TC, TG, HDL-C, LDL-C) and hs-CRP, lower levels of TC or LDL-C were also significantly associated with elevated hs-CRP. Secondly, we simultaneously evaluated the relationships among maternal 25(OH)D, lipid profile, and hs-CRP. Pregnant women with non-vitamin D deficiency have lower TC, TG, HDL-C, LDL-C, and hs-CRP levels compared with those deficient. Most importantly, based on stratified analysis, the significant association between high levels of TC, TG and elevated hs-CRP were only observed in pregnant women with vitamin D deficiency, but not the non-vitamin D deficiency. This study implicates the adequate vitamin D during pregnancy may inhibit high hs-CRP levels induced by hyperlipidemia.

To the best of our knowledge, some studies support the process of metabolic inflammatory, abnormal lipid profile levels may activate the systemic inflammatory response marker such as hs-CRP [16–19], which is similar to our findings in pregnant women. In addition, this is a study with the largest sample examining the role of vitamin D status in the association of lipid profile - hs-CRP during pregnancy, it was interesting that the intensity of the association steep ascended with the increasing levels of 25(OH)D up to 50 nmol/L and the strongest association was observed in 25(OH)D level above 50 nmol/L. This result further highlights that adequate vitamin D seems to enhance the effect on inhibition of lipid metabolism-inflammation. Given the adverse outcomes due to metabolic inflammation [20–22], taking vitamin D supplements during pregnancy for women with vitamin D deficiency may have clinical benefits in improving lipid and hs-CRP.

Our results are in agreement with previous results [23–26], which reported a significant correlation between abnormal lipid profile and elevated hs-CRP. However, there is no consensus on the relationship between lipids (TC, TG, HDL-C, or LDL-C) and inflammation. A cross-sectional study of 168 healthy school students found a positive correlation between TC/HDL-C ratio and hs-CRP levels [27]. Results in the general population of China indicate hs-CRP correlated positively with TG level, and negatively with the levels of HDL-C [28]. Another study showed that higher CRP levels correlated with lower levels of TC, LDL-C, and HDL-C in patients with rheumatoid arthritis [29]. Different findings might be explained by different factors such as ethnicity of the study population, geography, observation of gestational weeks and pre-pregnancy BMI. Notably, a non-linear relationship between lipid profile and hs-CRP may partly explain the inconsistency of the correlation. Our result suggested that lipid profile may have a dual biological effect, lipid metabolism indicators TC and LDL-C are beneficial to pregnant women within a certain range, exceeding this threshold may cause the level of inflammation marker hs-CRP to increase [30, 31]. Therefore, the dual biological effect of lipid metabolism indicators on inflammation during pregnancy is needed to further discussion.

Although observational studies showed that serum 25(OH)D level was negatively correlated with TC, and LDL-C, anther study have shown a positive correlation between vitamin D and lipids such as TC and TG in early pregnancy [12, 14]. It is worth noting that our results provided data that pregnant women with non-vitamin D deficiency had decreased lipid profile (TC, TG, HDL-C, LDL-C) compared with vitamin D deficient group. Inconsistent results may be related to vitamin D status. It suggests that adequate vitamin D may play an important role in lipid metabolism. And this report also concluded that the percentage of the pregnant women who had vitamin D deficiency has reached 75.6%, if vitamin D status has an effect on the lipid profile, this would be most easily observed that those with vitamin D deficiency combined with high lipid profile levels [32]. Unfortunately, prior studies assessing the effectiveness of vitamin D supplementation in reducing lipid profile found limited information and inconsistent results [33–35], most of the participants came from the same cohort, the sample size was small, different doses and the duration of the studies was different. Therefore, there is a need for the future of large-sample, multi-center, and high-quality interventional studies testing the effects of vitamin D supplementation on the lipid profile.

Additionally, a significant correlation between adequate vitamin D during pregnancy and low level of hs-CRP compared with those deficient group was found, and the association was previously observed in the general adult population [36], asymptomatic individuals [37], and neonates [10]. This result further supports the anti-inflammatory effect of vitamin D [38], and it seems to be important to avoid vitamin D deficiency during pregnancy. In this study, we further evaluated the potential biological pathway, and it supports the pathway of vitamin D deficiency may further enhance the systemic inflammatory response through aggravating dyslipidemia because of the significant positive association between lipid profile and hs-CRP was only observed among pregnant women with vitamin D deficiency. Although the mechanisms involved in this relationship are not understood, there is evidence that vitamin D limits the expression of microRNA in adipocytes in vitro and in adipose tissue in vivo through its impact on NF-κB signaling pathway [39]. These finding raised the possibility of vitamin D supplementation in pregnant women with vitamin D deficiency may be beneficial to improve metabolic inflammation.

This study elucidates the role of vitamin D status in the relationship between lipid profile and hs-CRP during pregnancy by using stratified analysis and nonlinear fitting models. The role of vitamin D in metabolic inflammation during pregnancy it was novel. Furthermore, an important strength is that this study relies on a prospective birth cohort study, the number of participants is relatively large, and confounding factors relevant to vitamin D status and lipid profile were strictly controlled to avoid confounding in statistical analyses. However, some limitations should also be mentioned in this study. First, there are no validated cutoff points to define abnormal lipid metabolism in pregnancy, a percentile cutoff is used to define elevated lipids, which might lead to misclassification, and the clinical significance of lipids is difficult to interpret. Second, due to the observational nature of our study, we cannot draw any causal relationship between vitamin D, lipid profile and inflammation. Third, we only measured hs-CRP and few lipid profile concentrations, which might not adequately represent the inflammatory or lipid metabolism of the participants. Finally, the generalizability of the results may be reduced by the fact that this study was conducted in one city of Hefei, and caution is necessary for generalizing the findings to other regions.

In conclusion, this study shows that there is a significant non-linear relationship between the lipid profile and the inflammation marker hs-CRP. Vitamin D may be a critical factor relevant to the association between lipid profiles and inflammation. Adequate vitamin D status during pregnancy may improve TC, TG, LDL-C levels and inhibit elevated hs-CRP induced by hyperlipidemia. In future research, the randomized controlled trial should be conducted to confirm the role of vitamin D supplementation on inhibition of inflammation through improving dyslipidemia.

## Supplementary information

**Additional file 1: Supplementary Figure 1.** Participants flow chart.

**Additional file 2: Supplemental Figure 2.** Estimated path analysis model showing the associations between serum 25(OH)D concentrations and hs-CRP.

## Data Availability

All data generated are included in this published article.

## Abbreviations

25(OH)D: 25-Hydroxyvitamin D
hs-CRP: High-sensitivity C-reactive protein
TC: Total cholesterol
TG: Triglyceride
HDL-C: High-density lipoprotein-cholesterol
LDL-C: Low-density lipoprotein-cholesterol
