# Supplementary material for: Vitamin D status affects the relationship between lipid profile and high-sensitivity C-reactive protein
Source: Nutr Metab (Lond). 2020 Jul 14;17:57. doi: 10.1186/s12986-020-00455-x (PMC7359462; doi:10.1186/s12986-020-00455-x)
Supplement: Supplementary file 2 — Additional file 2: Supplemental Figure 2. Estimated path analysis model showing the associations between serum 25(OH)D concentrations and hs-CRP. [file 12986_2020_455_MOESM2_ESM.pdf]

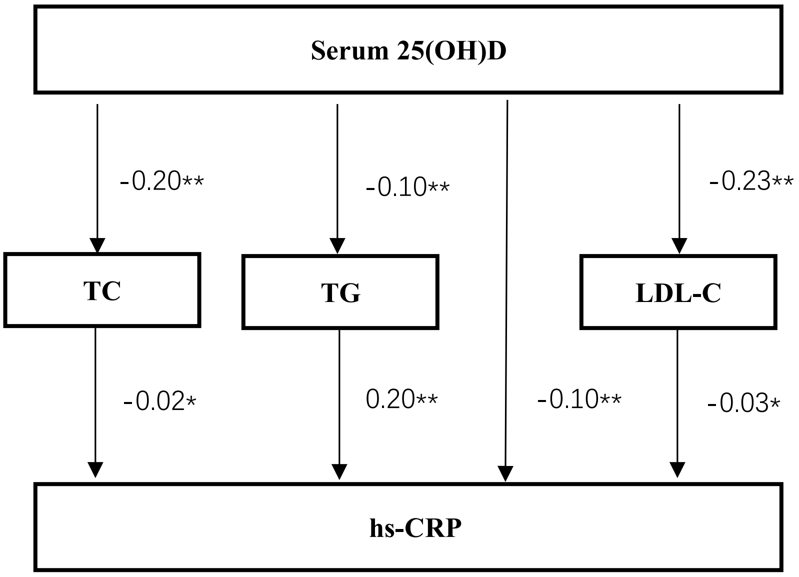

Supplemental Figure 2 Estimated path analysis model showing the associations between serum 25(OH)D concentrations and hs-CRP.

Hs-CRP is in milligrams per liter. The regression equations, with the potential mediators and hs-CRP as the outcomes, are represented by single-headed arrows. Coefficients with significance levels are presented beside each arrow (**P* < 0.10, ***P* < 0.05). The product of the coefficients along a compound path reflects the total weight of that path. All of the regression equations involved in the path analysis model were adjusted for sociodemographic characteristics (maternal age, education, income, season, parental diabetes and parental rheumatism), perinatal health status(pre-pregnancy BMI, systolic blood pressure and diastolic blood pressure), lifestyle(sedentary time, physical activities, paternal alcohol and smoking consumption, fish oil supplement, multivitamin supplement, milk intake, soy product intake and dessert intake). 25(OH)D, 25-hydroxyvitamin D; hs-CRP, high-sensitivity C-reactive protein; TC, total cholesterol; TG, triglyceride; HDL-C, high-density lipoprotein-cholesterol; LDL-C, low-density lipoprotein-cholesterol;
